# Context and Mutation in Gymnosperm Chloroplast DNA

**DOI:** 10.3390/genes14071492

**Published:** 2023-07-22

**Authors:** Brian R. Morton

**Affiliations:** Department of Biology, Barnard College, Columbia University, 3009 Broadway, New York, NY 10027, USA; bmorton@barnard.edu

**Keywords:** context dependency, plastome, genome evolution, mutation model

## Abstract

Mutations and subsequent repair processes are known to be strongly context-dependent in the flowering-plant chloroplast genome. At least six flanking bases, three on each side, can have an influence on the relative rates of different types of mutation at any given site. In this analysis, examine context and substitution at noncoding and fourfold degenerate coding sites in gymnosperm DNA. The sequences are analyzed in sets of three, allowing the inference of the substitution direction and the generation of context-dependent rate matrices. The size of the dataset limits the analysis to the tetranucleotide context of the sites, but the evidence shows that there are significant contextual effects, with patterns that are similar to those observed in angiosperms. These effects most likely represent an influence on the underlying mutation/repair dynamics. The data extend the plastome lineages that feature very complex patterns of mutation, which can have significant effects on the evolutionary dynamics of the chloroplast genome.

## 1. Introduction

The influence of the base composition of the surrounding nucleotides, or context, on mutation rates has been demonstrated in numerous genomes [1,2,3,4,5,6]. This effect can involve several flanking nucleotides, as shown in observations of the human genome in which the six surrounding six (the hexanucleotide context), three on each site, can influence mutation rates in the central base of the heptanucleotide [7]. One genome for which it is now well established that context has a significant influence on mutations is the plastid genome, or plastome [8,9,10]. In the angiosperms, mutations are significantly influenced by the surrounding hexanucleotide such that the rates of both transitions (ts) and transversions (tv) vary in a complex manner. However, despite this complexity, there are a few general results, among which are that the overall mutation rate is observed to vary by a factor of about 100 across hexanucleotide contexts, and the observed ts:tv varies by a factor of more than 200 [10,11]. Using context-dependent rate matrices generated from sequence comparisons, the expected equilibrium-base composition was calculated for the substitution dynamics within each context, and the equilibrium A + T content ranged from 36.4% to 82.8%, while the G-C skew and A-T skews ranged from −77.4 to 72.2 and −63.9 to 68.2, respectively [10].

These wide ranges in equilibrium-base composition illustrate how dramatically different the mutation process is across contexts. This could result from differences in a number of factors. Misincorporation by DNA polymerase may be dependent on context. In plants, there are two nuclear-encoded polymerases: Pol1A, which is involved and shows a high degree of fidelity; and Pol1B, which is probably responsible for the short replication associated with repair [12,13]. Forms of repair, such as mismatch repair, could also be context-dependent, and several nuclear-encoded mismatch-repair enzymes have been reported in chloroplasts [12], although the repair process in plastids varies across their development, with leaf tissue containing a significant amount of damaged chloroplast DNA that no longer acts as a coding molecule but, instead, as a degrading metabolic molecule [13]. Finally, the spontaneous mutation of epigenetically modified bases could also contribute to context variation in mutations, since known modifications occur in specific contexts. As our understanding of these processes in plastomes increases, we will be able to better assess contributions to context dependency and how contextual effects evolve across different lineages.

A dataset of pairwise sequence comparisons indicated that contextual effects are observed in plastomes across all the major lineages, not only flowering plants [8]. However, pairwise comparisons only allow general features of substitutions, such as overall ts:tv, to be analyzed. Here, we use a dataset of gymnosperm-plastome DNA to analyze sequence data from closely related triplets, which allows us to infer the substitution direction, as undertaken recently for angiosperms [10]. The dataset is insufficient to analyze more than the surrounding tetranucleotide, and even in this case, some G + C-rich contexts do not occur with sufficient frequency to provide data, primarily in noncoding DNA, but the analysis demonstrates that gymnosperm chloroplast DNA (cpDNA) shows strong context-dependent variation in substitution dynamics that are similar to the trends observed in angiosperms.

Despite the general similarities, there is one difference between gymnosperms and angiosperms which is of particular interest. A comparison of the noncoding (NC) sites with the fourfold degenerate (FFD) coding sites in angiosperms found a consistently higher rate of substitution at the FFD sites across contexts [11]. It was argued that the data were inconsistent with any effect of selection, and it was proposed that the difference may have arisen due to differences in the epigenetic modifications (or frequencies) between the coding and noncoding DNA. Such differences would have resulted in different mutation rates, since these can be affected by base modifications, such as the increased rate of C → T transitions at methylated CpG sites due to the deamination of the methylated cytosine [1,6,14,15,16,17], commonly referred to as the CpG effect. In this analysis, no such differences between NC and FFD sites were observed in the Gymnosperm cpDNA. If there is an effect of epigenetic modification on the differences from the angiosperms, then it does not appear to have occurred here. This suggests that an examination of the epigenetic modifications in the two lineages, and the differences between them, would be informative.

## 2. Materials and Methods

The analysis of substitutions followed the methodology described previously for the analysis of angiosperms, and a full description of all methods can be found in a previous study [10]. The RefSeq complete gymnosperm-chloroplast-genome sequences were downloaded from NCBI (www.ncbi.nlm.nih.gov/genome/browse#!/eukaryotes/ on 14 March 2019) and then parsed with the Biopython 1.76 [18]. Gymnosperm genomes were grouped into 17 triplets with an ingroup pair from the same family, and a randomly selected genome from outside that family as the outgroup. A list of the triplets is given in Supplementary Table S1 in the Supplemental Information. Alignments were performed as described in previous study [19]. For each triplet, an alignment was generated for each CDS and every intergenic region. The CDS alignments with more than 30 total gaps introduced and intergenic regions less than 70 nucleotides in length were all excluded from further analysis.

As described previously, in the angiosperm analysis, any site N_0_ can be considered as the central site of the pentanucleotide {N_2_ N_1_ [N_0_] N_1_ N_2_}, where N_1_, and N_2_ are the neighboring base pairs that compose the tetranucleotide context flanking N_0_. Substitution-count matrices were generated for each tetranucleotide context as in previous study [10,11]. Briefly, each matrix was 4 × 4 with rows and columns each arranged in the order {A, C, G, T}. For each aligned site the ancestral (*A*) and derived (*D*) states were inferred using the outgroup, and the M*_AD_* value of the matrix was incremented. Conserved sites, where *A* = *D*, provided the matrix diagonal values and allowed us to calculate rates, as described below. The CDS alignments were used to generate 128 4 × 4 tetranucleotide-context-dependent substitution matrices for FFD degenerate sites, with one matrix for each of the tetranucleotide contexts within which a FFD site could occur given the genetic code. Similarly, 256 4 × 4 tetranucleotide-context matrices were generated for NC sites. Since there was no evidence for strand asymmetry [20], the complementary count matrices were then combined to increase sample size of individual matrices [10]. In all analyses, only one from any complementary pair was utilized. The general A + T content for each tetranucleotide was measured using the AT Index (ATI), which is a measure of A + T content, with a higher weight given to the bases immediately flanking the substitution site [10].

Conserved sites within the alignments were included in the counts to make up the diagonals of the count matrices. This allowed us to generate rate matrices that represent Markov-transition matrices. The rate of substitution of a nucleotide within a specific context is simply the sum of the off-diagonal values in the appropriate row, and the overall rate within each context is the sum of all off-diagonal values divided by the matrix total. The stationary vector was also calculated for each rate matrix [21]. This vector represents the equilibrium-base composition to which a site in a given context evolves if the context is conserved. Although such conservation is not expected over time, differences between the stationary vectors of two contexts can be used as a measure of the differences in substitution pattern between contexts. To estimate sampling error for the A + T content of the stationary vector, the bootstrap method used by [13] was employed. Each resample generated a matrix with the same number of off-diagonals, or substitutions, in each row, with the substitutions drawn from the matrix row with replacement. The A + T content of the stationary vector from this resampled matrix was calculated and the average and standard deviation were determined for 1000 resamples. All calculations were performed using Python script, written by the author. Angiosperm data used in this study were taken directly from earlier studies [10,11].

## 3. Results

### 3.1. Context and Variation in Substitution Rate

**Figure 1:**
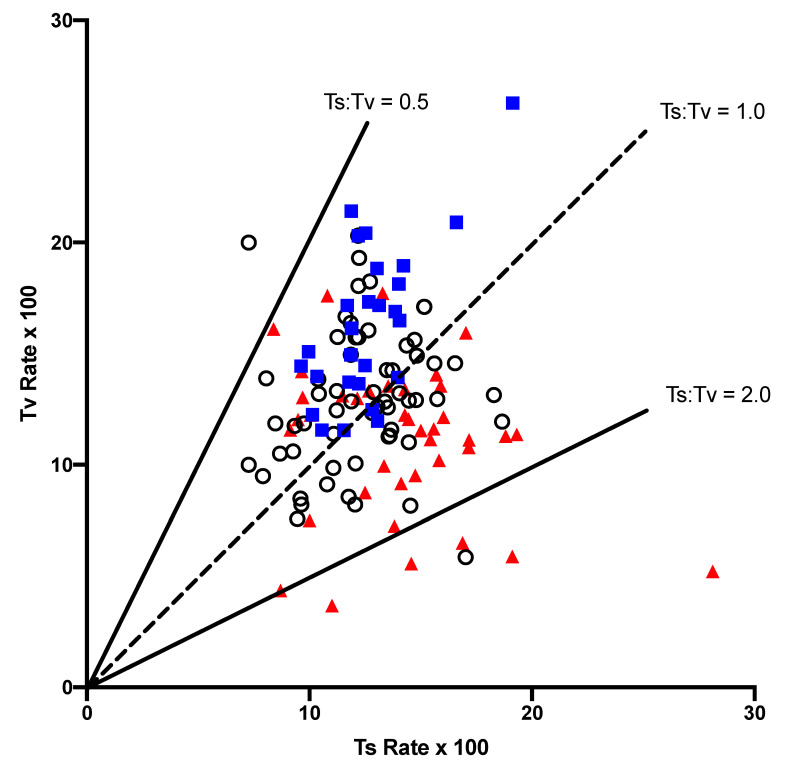
A comparison of the transition (ts) and transversion (tv) rates in each of the NC context-dependent matrices. Only matrices with at least 25 substitutions are included. Contexts that have an ATI greater than 9 (A + T-rich) are indicated as blue squares, while those with an ATI < 5 (G + C-rich) are indicated by a red triangle. All others are indicated by an open circle.

**Figure 2:**
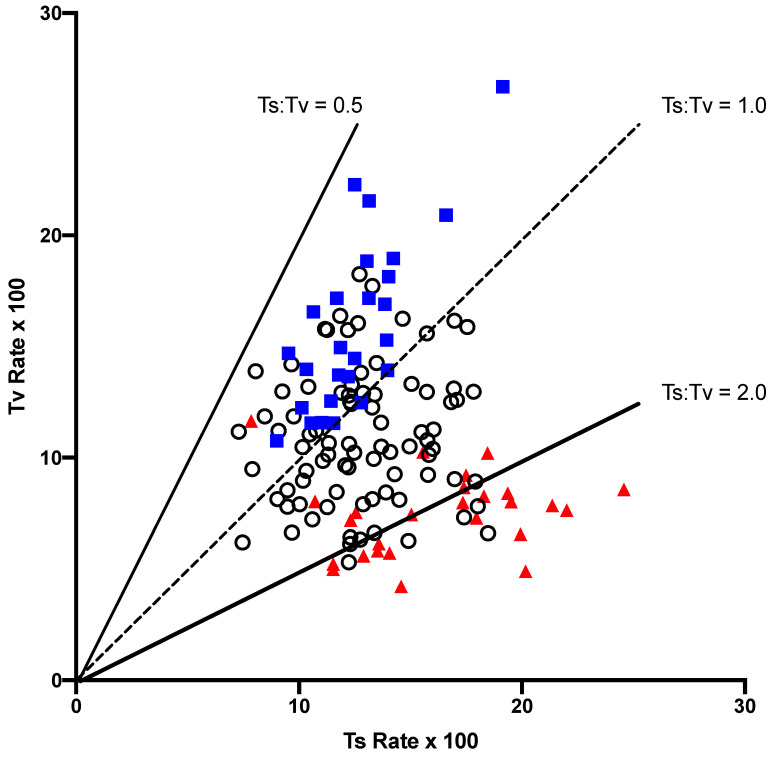
A comparison of the transition (ts) and transversion (tv) rates in each of the context-dependent matrices from the combined FFD and NC datasets. Only matrices with at least 25 substitutions are included. Contexts that have an ATI greater than 9 (A + T-rich) are indicated as blue squares, while those with an ATI < 5 (G + C-rich) are indicated by a red triangle. All others are indicated by an open circle.

Both the transition (ts) and the transversion (tv) rates varied across the tetranucleotide contexts in the gymnosperm NC regions (Figure 1). The two rates were not correlated (r^2^ = 0.0006), but there was a general influence of the A + T content of the flanking tetranucleotide. The contexts that were A + T-rich, as measured by the AT Index, (ATI, as defined in the Materials and Methods), tended to have higher transversion rates than the G + C-rich contexts, and there was a correlation between the rates of transition and transversion (r^2^ = 0.357). Another general feature was that the A + T-rich contexts generally had lower ts:tv values, lower than 1, while the G + C-rich contexts tended to have ts:tv > 1. Since most of the NC-substitution matrices that had fewer than 25 total substitutions, and were thus not included in Figure 1, were G + C-rich, the FFD and NC matrices for each context were combined (Figure 2). The combined dataset shows a very strong separation of the A + T-rich and GC-rich contexts in terms of the features described above for the NC dataset.

**Figure 3:**
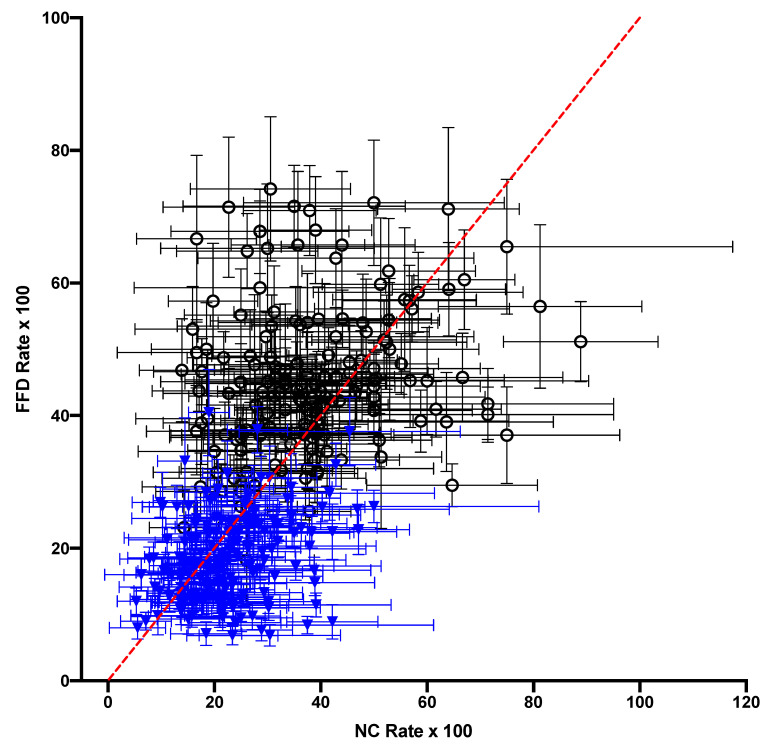
A comparison of the substitution rates from each nucleotide in each of the context-dependent matrices from the FFD and NC datasets (each point represents one row within one context-dependent matrix.) Substitutions from A or T are indicated by blue triangles, and those from G and C are represented as open circles. The line represents the equality line between the datasets.

**Figure 4:**
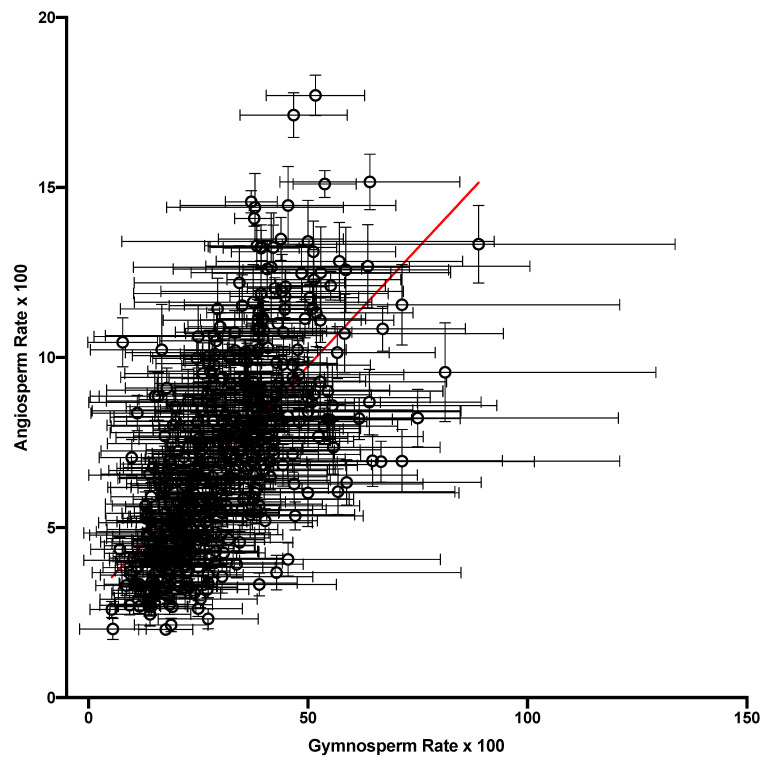
A comparison of the overall substitution rate of each of the context-dependent matrices from the NC datasets of gymnosperms and angiosperms. The regression lines is shown in red.

The substitution rates from each nucleotide were correlated across contexts between the FFD and NC datasets (Figure 3) in the gymnosperm, as was observed in the flowering plants. It was also observed that, as in the angiosperms, the substitutions from A and T generally had much lower rates than substitutions from G and C, which was not unexpected given the high A + T content of plastomes. However, unlike in the angiosperms, where the FFD sites had generally higher rates than the NC sites within the same tetranucleotide [11], in the gymnosperms, the data were equally distributed around the equality line, with FFD > NC for 50.9% of the points. This difference is striking, as discussed below. Although this difference between FFD and NC data was not shared between the lineages, the overall variation in the substitution rate across contexts was strongly correlated between the gymnosperm NC data and the angiosperm NC data reported by Morton (2022b) (r^2^ = 0.42, Figure 4). Overall, the data indicate that there was significant variation in the substitution rate, almost 100-fold, across the tetranucleotide contexts in the gymnosperm plastomes, and that these contextual effects were similar to those in the angiosperms.

### 3.2. Equilibrium Compositions of FFD and NC Sites

**Figure 5:**
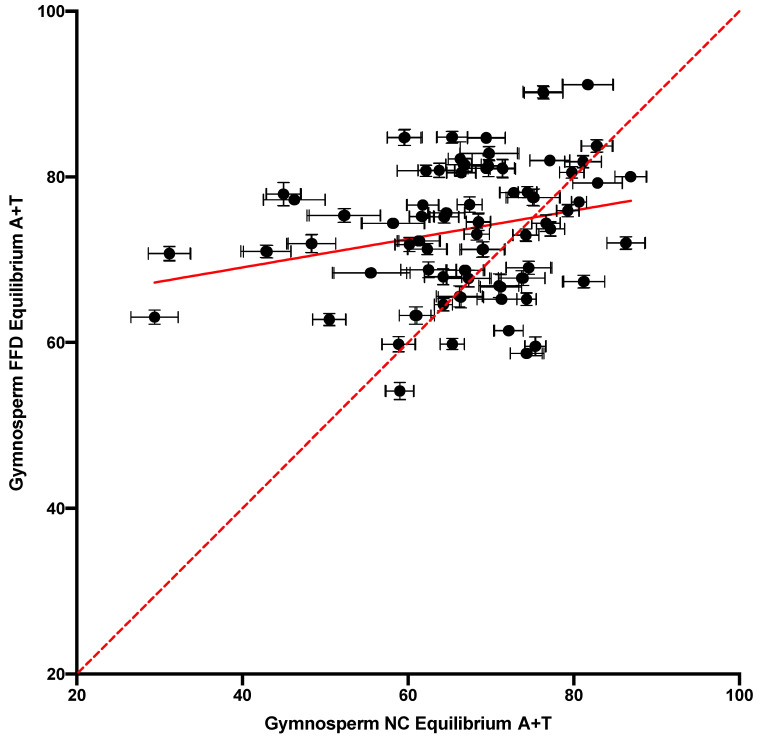
A comparison of the equilibrium A + T content of the stationary vectors of the context-dependent matrices from the FFD and NC datasets. The correlation is shown as a solid line, and the equality line is dashed.

**Figure 6:**
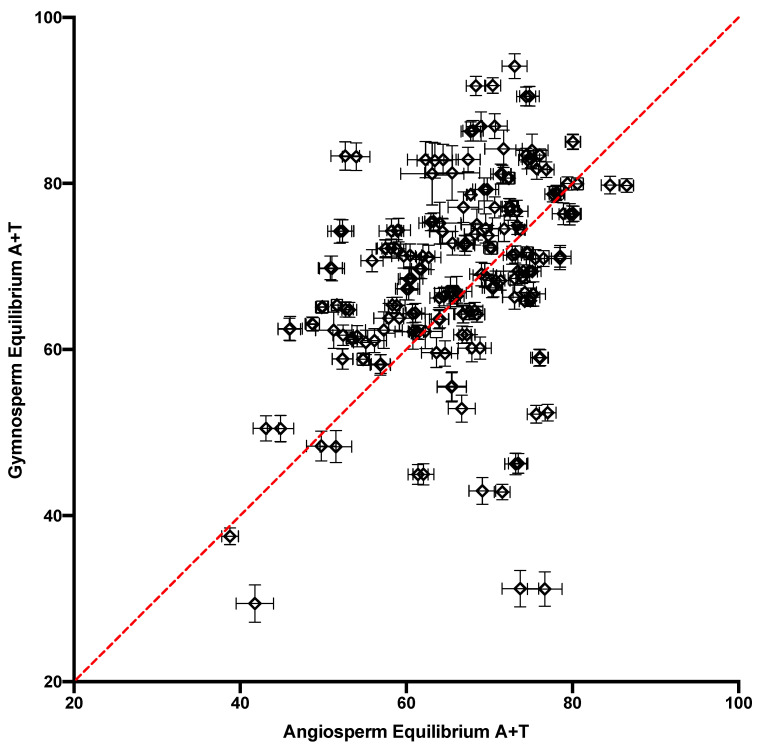
A comparison of the equilibrium A + T content of the stationary vectors of the context-dependent NC matrices from gymnosperms and angiosperms. The equality line is indicated.

Another way to assess variation in substitution dynamics across contexts is by using the equilibrium-base composition (i.e., the stationary vector) of their rate matrices. Although we do not expect the context of any site to remain invariant over time, the expected equilibrium composition is a useful measure of the substitution dynamics of sites in a given context, since differences in substitution dynamics appear as differences in the stationary state composition. The equilibrium A + T content across contexts is plotted for the NC and FFD sites in Figure 5. Both types of site showed significant variation, although the correlation was not strong (r^2^ = 0.09). However, they were roughly equally distributed around the equality line, with 56% of the contexts showing FFD > NC for equilibrium A + T, which yielded a Bayes factor of only 0.49. A comparison between the gymnosperm and angiosperm NC DNA demonstrated that the cpDNA in the two lineages showed similar substitution dynamics when measured according to this statistic (Figure 6). Overall, the data indicate that there was significant variation in the substitution process, and thus evolutionary trajectory, at the sites in different tetranucleotide contexts.

### 3.3. Epigenetic Effects

One fairly common contextual effect on mutation is the CpG effect in various genomes. This refers to an increased rate of transitions from Cs at CG dinucleotides as a result of the relatively frequent deamination of methylated Cs [6]. The data from angiosperm plastomes are consistent with a CpG effect, although a direct causal link with methylation is yet to be established [10]. Gymnosperms also display evidence of a CpG effect (Table 1). At both the FFD and the NC sites, the rate of C → T transition was about 23% higher at the CpG sites than in the other contexts. Although this observation is not itself evidence for an effect arising from CpG methylation, it is consistent with the phenomenon. Interestingly, the 23% increase is much lower than the 37% increase observed in angiosperms [10]. If the data in Table 1 are due to a real CpG effect, then they suggest that the methylation levels in this dinucleotide might be lower in gymnosperms than in angiosperms.

## 4. Discussion

The data in this analysis show that substitutions in gymnosperm cpDNA are strongly influenced by their tetranucleotide context in a manner that is similar to that observed in angiosperm cpDNA [10]. Context is associated significant variation in both transition and transversion rates, and the two are positively correlated across A + T-rich contexts. Since the same context dependency was observed at both NC (noncoding) and FFD (fourfold degenerate coding) sites, it is highly likely that the data resulted from the influence of flanking bases on the underlying mutation and/or repair processes. The alternative is that selection results in context dependency, but there is no good model for why selection in all of these degenerate sites would be dependent on the neighboring base composition, which would be necessary to give rise to the substitution patterns observed here. Instead, the influence of the flanking bases on processes such as misincorporation, mismatch repair, and spontaneous base mutation, including changes in epigenetically modified bases, is more likely to be responsible for the observations.

Although both the transition and the transversion rates varied across contexts, the two variables were not correlated, except in the A + T-rich contexts (Figure 1 and Figure 2). This indicates that any influence of context on misincorporation and/or mismatch repair effects different mutation types differently when the surrounding double-stranded molecule is more stable. One potential explanation is that different mismatch-repair systems might be affected independently by context, so that repair frequencies vary. A second potential explanation is that mismatch stability is influenced by context, which makes certain misincorporations more probable in each context, particularly in more stable helix environments. A third possibility, which is not mutually exclusive of the first two, is that different epigenetic modifications tend to occur in different contexts, giving rise to specific mutation rates, resulting from the spontaneous mutation of each particular modified base(s). Despite the lack of an overall correlation between the transition and transversion rates, we found that the A + T-rich tetranucleotide contexts tended to have higher transversion than transition rates, while the G + C-rich contexts showed the opposite relationship (Figure 2). This resulted in the context-dependent variation in ts:tv that was observed in earlier pairwise sequence comparisons [8].

The variation in substitution dynamics across contexts was also examined using equilibrium-base frequency. Using the observed substitution counts, including the conserved site counts, within each tetranucleotide context, a rate matrix was derived in the form of a Markov-transition matrix, for which the equilibrium vector could be calculated [21]. This represented the stationary (equilibrium)-base composition of any site evolving within that conserved context. Although we do not expect this to represent the actual stationary state of any site, since the context does not remain conserved over time, the differences in stationary base composition between any two contexts are useful measure of the overall difference in substitution dynamics, beyond the rate alone, between these two contexts. It is apparent in Figure 5 that there was a tremendous difference between the evolutionary trajectories of sites in different contexts in terms of stationary A + T content. This variation was correlated with the variation observed in the angiosperms (Figure 6). This demonstrates that sites in different tetranucleotide contexts have extremely different mutation properties, and this appears to be a general feature of the vascular-plant plastome.

One particularly interesting observation here was the equal distribution around equality in the comparison of the NC- and FFD-context-dependent rates (Figure 6). A previous analysis of angiosperm cpDNA found that FFD sites had consistently higher rates across contexts, and it was argued that the best explanation for this was that coding and noncoding regions may differ in terms of the frequency of certain epigenetic changes [11]. Since modifications, such as a methylated cytosine, can result in changes in mutation rate due to, for example, a high rate of deamination in a methylated cytosine, differences between regions in type and frequency of epigenetic modification may result in different mutation dynamics. If this is the explanation for the differences in rate between the coding and noncoding DNA in angiosperms, it suggests either that the epigenetic modifications responsible for differences do not occur in gymnosperm cpDNA, or that there is little difference between the modifications in coding and noncoding DNA. Since data consistent with a CpG effect were observed in the gymnosperms (Table 1), but at a lower level than in the angiosperms, it is possible that there were differences in epigenetic modification between the two lineages. This needs to be investigated in future studies for a better understanding of the underlying mutation process in plastomes.

The findings here on gymnosperm cpDNA provide further evidence for the complex context-dependent nature of mutations in plant cpDNA. Models of evolution, including those used to assess selection, need to account for this complexity if we are to fully understand plastome evolution. In particular, context dependency means that the composition of neutral sites is far more complex than is often assumed. Given the structure of the genetic code, the base compositions of neutral FFD sites in coding sites are not equivalent to the compositions of noncoding sequences, since the average context differs dramatically. Furthermore, the third position composition of different codon-degeneracy groups, or amino acids, does not share average contexts and, therefore, does not evolve to the same neutral base composition, meaning that different amino acids have biases towards different third-position bases, even in the absence of selection. This complicates predictions in relation to neutral composition, particularly codon-usage bias. For example, it has been shown that the assumption that FFD sites comply with Chargaff’s rules (that 1: A = T and G = C and 2: A + G = C + T hold true for one strand of a double-stranded DNA molecule) and that they also match the composition of noncoding regions, leading to faulty conclusions about codon usage and neutrality [19]. Context dependency can also influence phylogenetic reconstruction methods, particularly since it affects the probability of parallel changes at a site; it even affects protein evolution, since it biases processes such as the probability that an amino acid coded by a fourfold degenerate group will mutate to different twofold degenerate amino acids. This latter point can be illustrated by a comparison of valine (coded by GTN) and glycine (GGN). The different contexts of the third position of each mean that the two groups have different biases towards R vs Y in the third position. This, in turn, influences the probability of becoming a codon for aspartic acid (GAY) or glutamic acid (GAR) when there is a mutation in the second position. In addition to the implications for evolutionary analyses, the functional reasons underlying context dependency, including the possible role of epigenetic modifications, need to be investigated, as well as the full complexity of how context influences mutations in plastomes outside the gymnosperm and angiosperm lineages.

## Figures and Tables

**Table 1:** A comparison of the rate of C→T substitution in CpG contexts with the rate in other dinucleotide contexts for FFD (top) and NC (bottom) sites.

	Substitution ^1^			Substitution	
FFD	G→A	G→B ^2^		C→T	C→V
CG:	5606	12,488	CG:	6002	13,654
DG:	11,685	32,153	CH:	11,600	32,310
	OR = 1.24			OR = 1.22	
NC	G→A	G→B		C→T	C→V
CG:	874	2466	CG:	857	2427
DG:	4994	17,494	CH:	5012	17,482
	OR = 1.24			OR = 1.23	

^1^. Number of substitutions from C in the form of either a transition or a transversion, as indicated in the different columns, on either strand of DNA in the dinucleotide. The substituted base within the CG nucleotide is underlined in each case. ^2^. D = A, G or T (i.e., Not C); B = Not A; V = Not T; H = Not G

## Data Availability

All data are available at Dryad.

## Supplementary Materials

The following supporting information can be downloaded at: https://www.mdpi.com/article/10.3390/genes14071492/s1, Table S1: List of taxa triplets.
