# Supplementary material for: Context and Mutation in Gymnosperm Chloroplast DNA
Source: Genes (Basel). 2023 Jul 22;14(7):1492. doi: 10.3390/genes14071492 (PMC10378972; doi:10.3390/genes14071492)
Supplement: Supplementary file 1 [file genes-14-01492-s001.zip › genes-2496699-supplementary.pdf]

Table S1: List of taxa triplets

Thuja occidentalis, Cathaya argyrophylla, Pinus contorta[1]
Juniperus monosperma, Abies religiosa, Keteleeria davidiana
Agathis dammara, Picea abies, Cedrus deodara
Taxodium distichum, Pseudolarix amabilis, Nothotsuga longibracteata
Sequoia sempervirens, Pseudostuga sinensis, Larix decidua
Cathaya argyrophylla, Taxodium distichum, Glyptostrobus pensilis
Wollemia nobilis, Chamaecyparis formosensis, Chamaecyparis hodginsii
Cathaya argyrophylla, Sequoia sempervirens, Metasequoia glyptostroides
Pseudostuga sinensis, Thuja plicata, Thuja occidentalis
Taxodium distichum, Juniperus monosperma, Cupressus torulosa
Pinus contorta, Hesperocyparis benthamii, Callitropsis nookatensis
Sequoia sempervirens, Wollemia nobilis, Agathis dammara
Metasequoia glyptostroides, Araucaria bidwillii, Araucaria araucana
Agathis dammara, Ephedra sinica, Ephedra foeminea
Sequoia sempervirens, Juniperus monosperma, Chamaecyparis hodginsii
Pinus contorta, Wollemia nobilis, Araucaria bidwillii
Taxodium distichum, Hesperocyparis benthamii, Callitropsis nookatensis

1. Triplets listed as Outgroup, Ingroup 1, Ingroup 2
